# Minimal couple intervention to improve psychobiological stress resilience

**DOI:** 10.1111/bjhp.12799

**Published:** 2025-05-13

**Authors:** Corina Aguilar‐Raab, Martin Stoffel, Melanie Fischer, Monika Eckstein, Guy Bodenmann, Markus Heinrichs, Ulrike Ehlert, Beate Ditzen

**Affiliations:** ^1^ Department of Clinical Psychology, Interaction‐ and Psychotherapy Research, Institute for Compassionate Awareness and Interdependence Research and Practice IN‐CARE, School of Social Sciences University of Mannheim Mannheim Germany; ^2^ Institute of Medical Psychology, Centre for Psychosocial Medicine University Hospital Heidelberg, Ruprecht‐Karls‐University Heidelberg Heidelberg Germany; ^3^ Department of Psychology, Clinical Psychology Philipps‐University Marburg Marburg Germany; ^4^ Department of Psychology, Clinical Psychology for Children/Adolescents and Couples/Families University of Zurich Zurich Switzerland; ^5^ Department of Psychology, Laboratory of Biological Psychology, Clinical Psychology and Psychotherapy University of Freiburg Freiburg Germany; ^6^ Department of Psychology, Clinical Psychology and Psychotherapy University of Zurich Zurich Switzerland

**Keywords:** alpha‐amylase, cortisol, dyadic micro intervention, resilience, stress

## Abstract

**Objective:**

This study aimed at evaluating the effects of a minimal couple intervention focusing on positive aspects within the relationship (instructed partnership appreciation task; PAT) in daily life. We hypothesized a stress‐buffering effect of this intervention on perceived stress, salivary cortisol and alpha‐amylase.

**Methods:**

*N* = 40 couples were randomly assigned to either PAT or a no PAT (nPAT) condition. Self‐reports and saliva samples were assessed six times per day on five consecutive days. To account for couple interdependencies, multilevel modelling (MLM) approaches were used to test the effects of (a) group assignment (PAT vs. nPAT) and (b) practicing the PAT in everyday life (PAT group only).

**Results:**

Overall perceived stress was lower for women in the PAT group as compared with women in the nPAT group (*b* = −.380, *p* = .0098). *Within the PAT group*, daily positive interaction (PAT) significantly reduced cortisol (*b* = −.127, *p* = .02) and alpha amylase (*b* = −.122, *p* = .037). Sex‐specific analyses of within‐participants effects in daily life indicate that these results were driven by the men in the sample: Practicing the PAT led to a decrease in perceived stress (*b* = −.271, *p* = 001) and sCort (*b* = −.226, *p* = .006) in men, but not in women (all *p* > .05).

**Conclusions:**

The findings suggest that a minimal couple intervention can improve individual health‐related outcomes in a sex‐specific manner, and that effects depend on actually practicing the positive exchange in daily life.

**Trial Registration:**

The analysis of the present study is based on a sub‐sample (placebo group) of a larger neuropharmacological intervention and longitudinal trial ‘Oxytocin, Couple Interaction and Wound Healing’ (clinicaltrials.gov, identifier NCT01594775).

As social beings, humans thrive not only on social inclusion, but also on engaging in relationships that allow them to feel safe and supported. Close relationships are a significant attachment‐related resource. In particular, high relationship quality has positive effects on mental and physical health, as well as health behaviour (Roberson et al., 2018; Slatcher & Selcuk, 2017).

Well‐functioning couple relationships serve as an interpersonal resource. They are characterized by supportive and positive communication patterns, shaped by emotional self‐disclosure and intimacy as well as adequate coping skills (Bodenmann, 2005). Factors predicting nourishing relationships have been proposed in different models: These models focus on the components of the relationship itself, such as intimacy and/or on the couple's ability to deal with stressful events in a dyadic manner, for example, by providing mutual support to each other.

A fundamental precondition for the healthy relationship process is the ability to communicate verbally and non‐verbally about each other and the relationship, based on valuing each other's needs and their fulfilment (Davila et al., 2017; see also Berscheid, 2010). Beyond the positive characteristics that constitute a nurturing (romantic) relationship, the following models expand the resource‐oriented perspective by incorporating the competence to cope with stressors: The vulnerability‐stress‐adaptation model by Karney and Bradbury (1995) highlights that strengths and vulnerabilities not only affect the experience of stressful situations; they also influence a range of adaptive processes, just as these stressors themselves do, especially the degree of strengths and vulnerabilities present. These adaptive processes interact with aspects of relationship quality, which in turn predict relationship stability and functionality. Moreover, the Systemic Transactional Model (STM) by Bodenmann (2005) suggests that the way couples cope together (dyadic coping) with daily adversities, critical life events, or severe health issues can predict relationship satisfaction (Falconier & Kuhn, 2019), relationship stability as well as well‐being (Weitkamp et al., 2021). Emotion‐focused dyadic coping in particular has been shown to enhance positivity, intimacy, and attachment in couples.

The models discussed above highlight that internal and external stressors can jeopardize fulfilling relationships by hindering partners' ability to interact positively and adaptively. Among various coping strategies, relational coping involves communicative exchanges about positive experiences. For example, Horn et al. (2017) demonstrated that a lower frequency of sharing positive experiences and feelings by either partner correlated with a decline in relationship quality. This lack of capitalization (Gable & Reis, 2010) mediated the association between depressive symptoms and relationship quality.

Therefore, strengthening positivity in couples has received increased scientific interest in the last two decades, mainly in the context of positive psychology. For instance, a 4‐week intervention designed to boost excitement in committed relationships resulted in increased positive affect and relationship satisfaction. Follow‐up data suggested stable effects even 4 months after the intervention took place (Coulter & Malouff, 2013). Further, Woods et al. (2015) found that learning enthusiastic responding skills can enhance relationship well‐being. Teaching individuals to share positive experiences and respond actively and constructively boosts gratitude and relationship satisfaction with close friends or romantic partners. Another experimental study highlighted that incorporating gratitude into romantic relationships enhances positive emotions. Partners seen as highly responsive to gratitude expressions experienced better daily well‐being, including greater life satisfaction and positive emotions (Algoe & Zhaoyang, 2016). In a more recent pilot study, findings showed that incorporating intentional activities into couples' daily routines led to immediate improvements in positive indicators, such as positive emotions, mutual communication, interaction quality, and dyadic adjustment. Additionally, a brief weekly mindfulness practice may have further supported communication enhancement (Antoine et al., 2020).

Consistent with these findings, there is substantial theoretical and empirical evidence on the psychobiological effects of high relationship quality and positive social interactions (Robles et al., 2014), emphasizing the significance of relationship factors for individual health and well‐being. For instance, the Strength and Strain Model of Marital Quality and Health by Slatcher and Schoebi (2017) and Slatcher and Selcuk (2017) delineates the links between marital strain or strength and physical health outcomes, such as heart disease. These links are mediated by psychological (cognition, affect, behaviour) and biological mechanisms (endocrine, immune, cardiovascular, gene expression). The authors highlight two protective factors: (a) partner responsiveness, including caring and expressions of warmth, and (b) affective processes, such as emotional attunement, which reinforce positive emotions and strengthen connectedness. These protective factors are suggested to work through both implicit and explicit communication.

Fostering relationship quality in couples through improving partner responsiveness and positive sharing in daily life as stand‐alone or add‐on treatments may importantly increase the benefits of traditional psychological treatment approaches by their additional impact on physiological health outcomes (see also Aguilar‐Raab et al., 2018; Stanton et al., 2019).

Psychobiological outcomes are classically seen in stress‐sensitive measures of the hypothalamic pituitary adrenal (HPA) axis and the sympathetic nervous system (SNS). Indeed, the effector hormone of the HPA axis, cortisol (as measured from saliva samples, sCort) and more indirect outcomes of SNS activation, such as salivary alpha‐amylase (sAA) were suggested to mediate the linkage between marital quality and various clinical endpoints (Robles et al., 2014). During stress, both the HPA axis and the SNS enable a fine‐tuned response, reflected in an increase in adrenocorticotropic hormone, epinephrine, and norepinephrine. This results in increased cortisol, blood pressure, heart rate, and the enzyme alpha‐amylase. Both cortisol and alpha‐amylase can be non‐invasively assessed from saliva and interpreted as proxies for HPA axis and sympathetic activation in everyday life (Stoffel et al., 2021).

Consequently, sCort and sAA were interpreted as indicators of psychobiological stress responses (Ali & Nater, 2020) in couple research (Meyer & Sledge, 2020; Roberson et al., 2018). For instance, Meyer et al. (2019) investigated the association of perceived health, depressive symptoms, and relationship quality with diurnal cortisol in couples. Interestingly, women's higher depression scores as well as their lesser perceived physical health and relationship satisfaction were each linked to increased cortisol secretion in their male partners. This was not the case vice versa and not for men and women's own intra‐individual cortisol levels. Further, Kane et al. (2019) clarified that to cope with acute stress adaptively by means of emotion expression to one's partner post‐stress task (Trier Social Stress Test) in the lab was moderated by the trait‐level tendency to employ emotional approach coping: Participants with a higher degree of this coping approach showed larger sCort and sAA responses and more negative post‐task ruminative thoughts compared with those with a lesser tendency to cope by emotional approach coping. Interestingly, only those high in emotional approach coping benefited from partner support, as reflected by attenuated cortisol responses. Along similar lines, Meuwly et al. (2012) reported a significantly faster decline in cortisol associated with higher rates of positive dyadic coping in men as well as in securely attached women (Meuwly et al., 2012). Above this, it could be demonstrated that spontaneous expression of intimacy was associated with reduced cortisol responses to psychosocial stress in women and amplified cortisol recovery in both women and men during stress exposure in the laboratory (Ditzen et al., 2019). Similarly, intimacy in everyday life reduced cortisol levels in couples (Ditzen et al., 2008). On a broader level, Stoffel et al. (2021) have shown that the valence and quantity of social interactions in everyday life can considerably attenuate biological and psychological indicators of stress in everyday life. Furthermore, Bierstetel and Slatcher (2020) demonstrated that displays of affection as a positive interaction behaviour were associated with ‘healthier’ diurnal cortisol trajectories in couples, whereas exhibited contempt had the opposite association. In another EMA‐based study, positive interactions of healthy couples in daily life were negatively associated with carotid artery intima‐medial thickness – as an indicator of subclinical cardiovascular disease – while no associations were found between global ratings of relationship quality and intima‐medial thickness (Joseph et al., 2014).

Taken together, epidemiological data and basic research clearly associate positive couple interactions with individual health‐beneficial outcomes in the long term.

With the aim to translate these data into an intervention, we thus designed a standard instructed positive partnership appreciation task (PAT) to serve as a minimal couple intervention in everyday life.

The intervention aimed to cover three aspects: The first refers to directing both partners' attention to what is valuable and important to them and by providing a list of examples that might help to recognize and differentiate different aspects. Second, by focusing on positive experiences and positive resources within the relationship, both gratitude and positive expectations were hypothesized to be triggered. Finally, verbally expressing positive aspects to the other person (compliment sharing) as an appreciation was supposed to stimulate positive reciprocity between the partners (Algoe et al., 2013; Gottman, 1994; Lambert & Fincham, 2011). Basically, the intention was to create spillover effects from an abstract focus on relationship quality to concrete and explicit couple interaction. From this, a task (PAT) was developed, which instructed couples to go through and jointly discuss a list of positive aspects in their relationship (see details, below). In initial studies using the PAT in standard conditions in the laboratory, it was recently shown that such focus on positive aspects in the relationship improved relationship satisfaction (Warth et al., 2020) and interacted with intranasal oxytocin to alter immune markers in skin wounds (Ditzen et al., 2023).

We hypothesized that couples assigned to the PAT condition would improve stress resilience and psychobiological outcomes, as reflected in perceived stress reports, sCort and sAA on aggregated and daily levels, compared with couples in a control condition (non‐PAT ‐ nPAT). We assumed that both sexes would equally benefit from this intervention, but neither the less analyzed potential sex differences in response to the PAT.

## METHOD

### Participants

Healthy mixed‐gender, identified as monogamous and cohabiting couples were recruited via flyers, information brochures, internet ads, mailing lists of the University of Zurich, and social media (Facebook). Inclusion criteria were being between 21 and 45 years old and relationship duration between one and 15 years. Exclusion criteria were living with children, current or chronic physical or psychiatric illness (based on self‐report during phone screening), current medication (except for hormonal contraceptives), substance use (illicit drugs as tested via urinary multi‐drug test, alcohol intake on a daily basis, or smoking more than five cigarettes a day), body‐mass‐index <17 or >27. Women who did not use hormonal contraceptives participated during the early follicular phase of the menstrual cycle to minimize effects of the cycle on HPA axis and ANS activity and make groups comparable.

The present report is embedded in a larger project. The mother longitudinal neuropharmacological intervention study was pre‐registered at clinicaltrials.gov (identifier NCT01594775). Following an initial power calculation including sex and group assignment and pharmacological treatment as independent variables, same‐gender couples were excluded from study participation in the mother trial and, therefore, not included in the present analysis.

In total, 80 couples (*N* = 160 individuals) were included in the larger project. The sub‐sample used in the present analysis consists of the 40 couples (*n* = 80 individuals) who were in the placebo condition. They were randomized into two groups: One group was instructed in a short verbal positive interaction task they practised in the lab and then were asked to practice this interaction task up to 2 times in their everyday lives during the assessment period (Partnership Appreciation Task Group, PAT: *n*_individuals_ = 38; nPAT Group *n*_individuals_ = 42). Based on the initial phone screens, couples were stratified with half of the women in each group using hormonal contraception; the other half were naturally cycling. Couples reported overall high relationship quality (assessed via PFB, Hahlweg, 1996) with women reporting higher relationship quality than men (*M*_women_ = 74.05 ± 6.61, *M*_men_ = 71.58 ± 8.07, *t(39)* = 2.090, *p* = .043). The two groups PAT/ nPAT did not differ in relationship quality (*F* = .772, *p* = .283). Sample characteristics and descriptive data of control and outcome variables are depicted in Table 1.

**TABLE 1:** Sample characteristics and descriptive data by group (PAT/nPAT).

	PAT				nPAT			
	Male		Female		Male		Female	
	*n*	M (SD)	*n*	M (SD)	*n*	M (SD)	*n*	M (SD)
Age (years)	19	27.74 (4.45)	19	26.00 (2.73)	21	29.62 (6.00)	17	28.18 (6.89)
Body‐mass‐index (kg/m^2^)	19	23.83 (1.99)	19	20.84 (2.14)	21	24.11 (1.43)	17	21.02 (1.93)
Perceived stress^a^	19	1.09 (.48)	19	1.13 (.51)	21	1.14 (.44)	21	1.55 (.40)
Alpha amylase (U/mL)	19	90.12 (62.67)	19	68.39 (47.74)	21	111.76 (75.51)	21	110.94 (65.30)
Cortisol (nmol/L)	19	6.82 (2.21)	19	7.32 (1.99)	21	7.61 (2.15)	21	8.61 (3.13)
Intake of meal^b^	19	3.23 (.73)	19	3.22 (.75)	21	3.17 (.57)	21	2.91 (.69)
Intake of drink^c^	19	3.30 (.67)	19	3.39 (.85)	21	3.32 (.92)	21	2.97 (.84)
Day activity^d^	19	1.89 (.72)	19	2.51 (.92)	21	2.62 (.94)	21	2.09 (.94)
Sleep quality^e^	19	6.14 (1.21)	19	6.20 (1.41)	21	2.53 (1.17)	21	6.13 (1.17)

		*n* (%)	*n* (%)	*n* (%)	*n* (%)
Hormonal contraceptives	Yes	—	9 (47%)	—	9 (43%)
	No	—	10 (53%)	—	8 (38%)

		Events (%)	Events (%)	Events (%)	Events (%)
Sleep problems^f^	Yes	7 (7%)	17 (18%)	4 (4%)	12 (11%)
	No	88 (93%)	76 (80%)	100 (95%)	91 (87%)
Practising PAT in daily life^g^	Yes	30 (32%)	33 (35%)	—	—
	No	58 (61%)	60 (63%)	—	—
Intake of caffeine^h^	Yes	242 (21%)	242 (21%)	286 (23%)	286 (23%)
	No	693 (61%)	693 (61%)	753 (60%)	753 (60%)

*Note*: For all repeatedly measured (non‐dichotomous) variables, person means are reported here. For all dichotomous variables, the total sum of responses/measurements for each specific subgroup is reported. Here “*n*” is the number of participants in the respective subgroup (hormonal contraceptives). “Events” shows the number of participants in the subgroup multiplied by the number of potentially possible measurements for each participant (i.e., once per day for sleeping problems or practising the PAT and five times per day for caffeine intake). Missing observations are missing values (e.g., because of technical issues with the data assessments or missed prompts).

^a^ *Please indicate how you feel at the moment*. (visual analogue scale: 0 = relaxed to 4 = stressed).

^b^ *What have you eaten since the last occasion?* (visual analogue scale: 0 = small to 9 = big meal).

^c^ *What have you been drinking since the last occasion?* (visual analogue scale: 0 = small to 9 = large quantity).

^d^ *How physically active have you been since the last occasion?* (visual analogue scale: 0 = little active to 9 = very active).

^e^ *How well did you sleep?* (visual analogue scale: 0 = very bad to 9 = very good).

^f^ *Have you had trouble falling asleep?* (Total number of days on which all participants in the specific subgroup reported sleeping problems.)

^g^ *Have you practised the standardized interaction today?* (The assessment took place once per day at the last prompt. Thus, the total number of days on which all participants in the specific subgroup practised the PAT in their daily life's is reported here.)

^h^ *Have you ingested caffeine?* (Total number of occasions at which all participants in the specific subgroup reported to have consumed caffeine since the last occasion.)

All participants provided written informed consent. Each couple received 500 CHF (about 510 USD) for study completion. The study protocol was approved by the local ethics committee of the Canton of Zurich, swissmedics, and the study was monitored by the Clinical Trials Centre Zurich. The research was conducted in accordance with the Declaration of Helsinki. All data, analysis code, and research materials can be obtained from the corresponding authors and are accessible for research on Heidelberg University's data repository website (https://heidata.uni‐heidelberg.de/).

### Procedure

After telephone screening for eligibility, couples were invited to an instruction session at the laboratory. To match the EMA iPod programming to participants' individual daily routines, participants provided information on their general awakening times (for the five consecutive days of EMA). During the laboratory appointment, participants provided urine samples to rule out drug consumption and pregnancy and completed questionnaires on relationship characteristics and demographics. Subsequently, participants were instructed on how to use a pre‐programmed (iDialogPad, G. Mutz, Cologne, Germany) iPod touch® as well as ambulatory saliva sampling using the SaliCap® system (IBL, Hamburg, Germany).

Couples were asked to complete the EMA on five consecutive days during a normal week in their lives and not to spend any night during the assessment period apart from each other. Measurement time points were prompted by iDialogPad directly after awakening (T1; M = 7:47 am), +30 min (T2; M = 8:21 am), +2.5 h (T3; M = 10:30 am), +8 h (T4; M = 4:03 pm), +12 h (T4; M = 7:26 pm), and at bedtime (T6; M = 11:43 pm). At each time point, participants provided saliva samples. They were asked not to brush their teeth prior to the first two saliva measures in the morning in order not to contaminate the saliva samples with blood. The data suggest that compliance with the EMA protocol was high, given that, on average, (a) 97.21% (*SD* = 5.36%) of all possible values for data derived from saliva samples (sCort and sAA) are available and (b) 97.68% (*SD* = 4.35%) of all possible occasions where data from saliva and self‐reports on perceived stress were assessed resulted in valid data.

### Measures

#### Perceived stress

Perceived momentary stress was assessed at T3–T6 (e.g., Wuttke‐Linnemann et al., 2019; Klein et al. 2016) with the item ‘Please indicate how you feel at the moment’ and a response scale ranging from 0 = ‘stressed’ to 4 = ‘relaxed’. To enhance interpretability, the item was reversed prior to statistical analyses. Adherence with subjective momentary measures was very high: Out of 1600 possible values for perceived stress, 22 values were missing.

#### Biological measures

SaliCaps® (IBL, Hamburg, Germany) were used for the collection of saliva via passive drool either using a straw or by salivating directly into the tube at T1–T6. In order to ensure correct labelling, participants had to enter the number of the SaliCap they used into the iPod at each prompt. Participants were instructed to then keep their samples in their refrigerators at home until returning them to the laboratory. Samples were frozen at −20°C until analyses for no more than 6 months. sCort and sAA were analysed at the Biomarkers Lab at the Institute of Psychology, University of Zurich. sCort was analyzed using a commercially available competitive luminescence immunoassay (IBL, Hamburg, Germany). Concentrations are given in nmol/L. For the measurement of sAA, a kinetic colorimetric test and reagents from Roche (Roche Diagnostics, Mannheim, Germany) were used. Results on sAA concentrations are reported in U/mL. The intra‐assay coefficient of variation was below 10% for both. Out of 2400 potential values for these parameters, four were missing for sCort and 67 were missing for sAA.^1^

#### The intervention: Partnership appreciation task (PAT)

Couples were randomized to a positive interaction condition (Positive Interaction Group), the Partnership Appreciation Task PAT (Warth et al., 2020), or a control condition (nPAT). The PAT was conceptualized as an instructed positive appraisal of the relationship and personal characteristics of each partner. In the lab, couples received a list of 23 topics, which describe important domains of romantic relationships (e.g., social support, etc.; see Supplement S1). They were asked to rate each of these topics on a 4‐point Likert scale (0 = does not apply to our relationship, 4 = is a frequent/ important aspect in our relationship) and to talk about some of them for 10 min regarding their own relationship. Above all, they were instructed to talk positively about the partnership, that is, to name characteristics of the other person that they particularly appreciate. They could amend further positive aspects in case any deemed them missing in the list. During this instructed task, couples were videotaped to ensure adherence to the task. In the control condition (nPAT), couples spent 10 min waiting without the instruction to interact in a specific manner, again being videotaped. During the laboratory visit, mood was assessed six times using a visual analog scale (1–10).

Couples in the intervention group then were instructed to practise the PAT up to two times during the coming week and to document any new or important positive aspect in their relationship with the iDialogPad in their iPods. On each day of the study, they were further asked to report whether they had practised the PAT or not. Couples in the control group received no such instruction.

### Statistical analyses

All analyses were performed in the statistical environment *R* (R Foundation for Statistical Computing, 2020).

Multilevel models (MLM) were used to account for the nested structure of the dyadic data (following Atkins, 2005). Measurement within days (L1) were treated as nested in participants (L2) which were treated as nested in dyads (L3). MLMs were fitted via ‘nlme’ package (Pinheiro et al., 2020) with a restricted maximum likelihood method (REML). The first two data points provided during the post‐awakening period (i.e., the first 45 of each day) were excluded because there were no assessments of perceived stress or important controls for sCort or sAA at the first two sampling occasions. All variables were screened for their distributional properties. In this process, all biological data, but not data on perceived stress, were found to be positively skewed. Consequently, for the MLMs, these variables were transformed to the natural logarithm. Outliers beyond three SDs of the mean were excluded before fitting any of the models.^2^

To control for their effects, all covariates, except those indicative of time passed since awakening in minutes, were centred on their grand mean. Time within days was entered to detrend the circadian rhythms/within day fluctuations of the dependent variables (see Stoffel et al., 2021; Wang & Maxwell, 2015). The effects of covariates should not be interpreted because they confound the effects of within‐ and between‐participant variation (Wang & Maxwell, 2015). To fit baseline models, all covariates were included together with random intercepts on L2 and on L3. The effects of time within days were plotted to identify possible nonlinear trajectories and were added as fixed whenever they improved the model fit; these models were fitted using ML estimations. Random intercepts were always included to minimize standard errors (Bliese & Ployhart, 2002). Given that variables varied across L3, they were separately tested as random slopes: We built one model for each possible random slope, which was then compared with the baseline model. Random slopes were kept in the final model whenever they improved the model fit. Model comparisons were conducted using likelihood ratio tests and the Bayesian information criterion (BIC; to avoid overfitting). Random effects were only tested on L3, given that there are only two degrees of freedom on L2 (see Atkins, 2005). Each final model was graphically assessed for violations of central model assumptions regarding their residuals. There were no severe violations of these assumptions in any of the models reported. For models focusing on within‐participant effects, pseudo‐*R* ^2^ measures were calculated following the approach from Nakagawa et al. (2017), which is an extension of the approaches by Nakagawa and Schielzeth (2013) and Johnson (2014), using the function ‘r.squaredGLMM’ from the ‘MuMIn’ package (Barton, 2020). Only conditional pseudo‐*R* ^2^ values are reported, since they are indicative of the variance explained by the entire model. For each final model, the total number of observations, a list of all covariates, as well as time trends and random slopes, which lead to an optimized model fit, are presented in separate tables (see Tables in Supplement S3).

#### Between‐dyad effects of the intervention in everyday life

Between‐dyad effects of the intervention (group assignment: PAT vs. nPAT) were conducted using data from all participants. MLMs were used to test the association of PAT (coded as factor; nPAT/PAT), as the focal predictor on L3,^3^ with the three dependent variables.

#### Within‐participant effects of practicing the PAT in everyday life

Within‐participant analyses were conducted only within the PAT group. To obtain pure within‐participant effects of practising PAT in everyday life (treated as factor with 0 = no and 1 = yes) on the three outcomes, together with PAT, the untransformed person means of PAT were added to the models (Yaremych et al., 2023). The effect of these person means (days on which participants in the PAT condition reported to have practised the interaction) of PAT can be described as ‘contextual effects’ of the PAT (Yaremych et al., 2023); however, these effects were not of interest and, thus, are not further interpreted. Of note, there was only one measure of ‘practising the PAT’ per day per participant. Following from this, within‐participant effects can be interpreted changes in the outcomes on days when a person practised the PAT as compare to days when they did not (i.e., the effects can be interpreted from day to day instead of from measurement occasion to measurement occasion; see Supplement S2 for multilevel equations).

### Sex‐specific analyses

To shed light on possible sex‐specific within‐participant and between‐dyad dynamics, we also fitted multivariate two‐level models for distinguishable dyads (see Atkins, 2005; Laurenceau & Bolger, 2005). In these models, the three‐level structure of the data (measurements nested in persons nested in dyads) is represented in two‐level models (measurements nested in dyads), where person‐specific intercepts and slopes are estimated (i.e., for each partner within the dyad). Besides the differences given by the multivariate structure of the model, the general approach to fit the models was identical to the approach described for the three‐level models (see above).

## RESULTS

### Descriptive data

Table 1 shows that, on a descriptive level (Means and SDs), perceived stress, sCort and sAA were lower in the PAT condition, compared with the nPAT condition; however, there were sex‐specific patterns.

### Results of multilevel models

#### Group assignment: Between‐dyad and sex‐specific effects of the intervention in everyday life

Results of three‐level MLM suggest that overall, PAT (coded as factor; PAT/nPAT) was not significantly associated with perceived stress (*b* = −.198, *p* = .08; model 1), sCort (*b* = −.197, *p* = .125; model 2), or sAA (*b* = −.32, *p* = .191; model 3). Tables S1 to S3 in Supplement S3 summarize models 1–3; the corresponding multilevel equations can be found in Supplement S2.

##### Results of sex‐specific analyses

**FIGURE 1:**
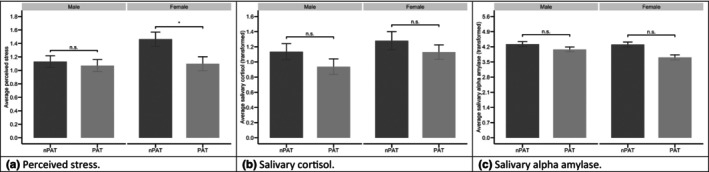
Average levels (descriptive values) of perceived stress (a), salivary cortisol (b) and salivary alpha‐amylase (c) grouped by group assignment (nPAT/PAT) and sex (male/female). The error bars indicate the 95% confidence interval for the mean. The indicators of significance refer to multivariate multilevel models which tested the effects of group assignment, separated for males and females (between‐dyad effects, tested in models 4–6); *Indicates *p* < .05 while n.s. indicates that the effect was not significant. The figure was fitted using data from models 4 to 6.

Sex‐specific analyses (two‐level dual intercept MLM) showed that women, but not men, in the PAT group reported lower perceived stress on average (as compared with women in the nPAT group; *b* = −.380, *p* = .0098; model 4. Table S4 in Supplement S3). No sex‐specific effects of group assignment on sCort or sAA were found (all *p* > .05; models 5 and 6, Tables S5 and S6 in Supplement S3). Multilevel equations for models 4–6 can be found in Supplement S2. Using all observations from within the respective models, Figure 1 visualizes sex‐specific patterns for the effects of group assignment on perceived stress, sCort, and sAA.

#### Practising the PAT in everyday life: Within‐ participant effects

Within the PAT group, results from three‐level MLM indicate that the fixed effect of practising the PAT on perceived stress was not significant (*b* = −.184, *p* = .083; model 7, Table S7, Supplement S3; multilevel equation in Supplement S2). However, Pseudo‐*R* ^*2*^ increased from 32.33% (random intercepts only) to 36.35%, indicating that between‐dyad variations in the effects of practising the PAT additionally explained approximately 4% of the variance. Above this, the three‐level MLM suggests significant within‐participant effects of practising the PAT on sCort (*b* = −.127, *p* = .02; model 8, Table S8, Supplement S3; multilevel equation in Supplement S2) and on sAA (*b* = −.122, *p* = .037; model 9, Table S9 in Supplement S3; multilevel equation in Supplement S2). Pseudo‐*R* ^*2*^ measures for these models indicate that they explain 54.56% of the variance in sCort and 63.08% of the variance in sAA. By entering the PAT predictor into the models, in comparison to their baseline models, models with the focal predictors increased the Pseudo‐*R* ^*2*^ values by 1.35% for perceived stress and by 0.62% for sCort as well as for sAA. In addition, for the model predicting perceived stress, there was a significant random slope of PAT on L3 (*Χ* ^2^(2) = 13.5, *p* = .001).

##### Results of sex‐specific analyses

**FIGURE 2:**
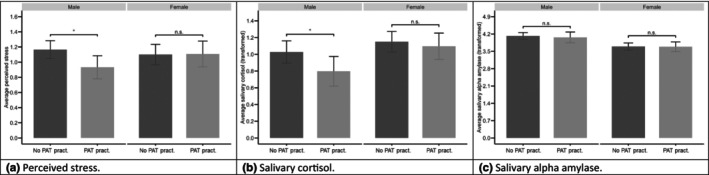
Average levels (descriptive values) of perceived stress (a), salivary cortisol (b) and salivary alpha‐amylase (c) grouped by whether a person in the PAT group practised the PAT in everyday life (PAT pract.) or not (No PAT pract.) and sex (male/female). The error bars indicate the 95% confidence interval for the mean. The indicators of significance refer to multivariate multilevel models which tested the effects of practising the PAT in everyday life, separated for males and females (within‐participant effects, tested in models 10–12; *Indicates *p* < .05 while n.s. indicates that the effect was not significant). The figure was fitted using data from models 10 to 12.

Sex‐specific analyses of the within‐participants effects in everyday life (two‐level dual intercept MLM) suggest that the results in the intervention group were driven by the men in the sample: Practising the PAT in everyday life lowered perceived stress (*b* = −.271, *p* = 001; model 10, Table S10, Supplement S3) and sCort (*b* = −.226, *p* = .006; model 11, Table S11, Supplement S3) in men, but not in women (all *p* > .05; models 10 and 11, Tables S10 and S11, Supplement S3). No within‐participant effects on sAA emerged in the two‐level dual intercept MLM (*p* > .05; model 12, Table S12, Supplement S3). Multilevel equations for models 10–12 can be found in Supplement S2. Using all observations from within the respective models, Figure 2 visualizes sex‐specific patterns for the effects of practising the PAT in everyday life on perceived stress, sCort, and sAA.

## DISCUSSION

In this study, we aimed to investigate the effects of the instructed positive partnership appreciation task (PAT) as a minimal couple intervention to improve stress resilience in daily life, according to positive psychology interventions (Bradbury & Bodenmann, 2020). The PAT was developed based on large epidemiological data on positive health outcomes in satisfied couples. Couples were instructed to focus on specific positive aspects in their own relationship and the partner (Warth et al., 2020), an approach that is related to the evidence‐based behaviour exchange techniques (Jacobson & Margolin, 1979).

Following this intervention, on a descriptive level overall perceived stress, sCort and sAA were lower in the PAT condition. Statistical analyses using MLMs showed that women within the PAT group reported significantly lower levels of perceived stress than women in the nPAT group. This suggests that average levels of perceived stress in daily life could be reduced as a result of taking part in the PAT in women, but not in men. No significant group differences emerged for sCort or sAA on an overall level.

On a more fine‐grained daily level focusing on within‐participant variations within the PAT group, participants had lower sCort and lower sAA outputs on days when they actually practised the PAT with their partner, compared with days on which they did not practise. Regarding perceived stress, the effect of practising the PAT was not significant when between‐dyad variations were considered, which explained a considerable proportion of variance. Overall, this suggests that the effect of practising the PAT on perceived stress (but less so on sCort or sAA) seems highly dyad‐specific. This could indicate that the effects of an instructed positive exchange on perceived stress depend on a variety of couple characteristics. Indeed, previous studies have shown the effects of interventions for couples could depend on factors such as other social relationships or economic factors (see Bradbury & Bodenmann, 2020 for a review).

Sex‐specific results from the multivariate two‐level models showed that for men only, perceived stress and sCort^4^ were lower on days when dyads practised the PAT. No sex‐specific significant effects of practising the PAT emerged regarding sAA. From a statistical point of view, the results suggest that (a) effects are, in general, more pronounced on the within‐participant level and that (b) these effects are also sex‐specific.

Overall, the results are in line with the stress‐buffering effects of positive social interactions. Being actively engaged in positive interactions with the partner might reduce subjective and biological stress below the levels usually experienced by a person, especially in men. Also, results suggest that on a subjective level, women, but not men, generally benefited from taking part in the intervention.

In everyday life, negative interactions and social conflicts carry a lot of weight given their threat value. This makes it all the more crucial to provide evidence‐based psychological interventions fostering positive interactions as part of preventive and clinical efforts to help cultivate important social relationships.

Couples living together and sharing a substantial amount of time in their lives have a mutual impact on experiences and health behaviour: the way they perceive, think, feel, and act – even the psychobiological patterns of partners are synchronized and aligned to each other (e.g. Coutinho et al., 2019). Hence, it seems advisable to establish dyadic micro interventions – couple interventions that are easy to teach, do not require much effort to be integrated into daily life, and could be incorporated into counselling and clinical treatments for psychological disorders. Such efforts might increase self‐efficacious interpersonal relationship building in the interest of appropriate co‐regulation and support the development of skills that promote togetherness in intimate relationships and, in turn, contribute to various health outcomes (e.g., Chang et al., 2022).

So far, relatively little attention has been paid to strategies to maintain the feeling of togetherness in everyday interactions by means of enactments of positive verbal and non‐verbal communication patterns (e.g., Zhou et al., 2022). This study suggests that (re‐) directing attention to valuable aspects of the partner and the benefits of the relationship, even when employed as an isolated intervention, could potentially guide couples to a more resilient way of interrelating (Adair et al., 2018; Allen et al., 2021; Lambert & Fincham, 2011).

Notably in the present study, the intervention exclusively focused on positive aspects within the relationship and about each other. No problem‐solving task or analysis of difficult aspects in the relationship was included. It could be hypothesized that in the absence of a problem‐solving component, explicit praise and emphasis on positive aspects of the relationship may influence deep‐seated cognitions related to social identity, evaluations of the self within social contexts, and perceived social worth. This, in turn, could lead to stress‐reducing effects for the individual, warranting further investigation.

More specifically, partner responsiveness and affective processes which are addressed in the above‐mentioned Strength and Strain Model of Marital Quality and Health seem to be key elements (Slatcher & Schoebi, 2017; Slatcher & Selcuk, 2017): Attachment‐based processes facilitate mutual security between partners, while partner responsiveness involves demonstrating respect through caring, understanding, and validation. Fine‐tuning interpersonal awareness and affective attunement enhances partners' orientation towards each other, fostering emotional closeness and intimacy. Social affiliation, identification processes, attachment styles, and other personality traits significantly contribute to this dynamic interaction (Rimé et al., 2020). Future studies should delve into these nuanced mechanisms.

We found sex‐specific effects of the intervention on sCort and perceived stress in this study. These results suggest that while women reported lower perceived stress in the PAT condition, men who actively practised the PAT benefitted considerably more from the exchange of positive attributes in their relationship during their daily life routines. This could be due to various reasons associated with the characteristics of these couples such as length of relationship, individual and partner stress levels, etc. However, some studies suggest gender differences, where women are on average more sensitive to negative couple interaction (Kiecolt‐Glaser & Newton, 2001), and are more affected by and respond stronger to their partners health problems and burdens than vice versa (Kiecolt‐Glaser & Wilson, 2017). Although it appears that the relationship between perceived stress, cortisol, and conflict behaviour appears to be similar for men and women, there is nevertheless also evidence that average cortisol rates were higher in men versus women in a study of marital conflict behaviour (Shrout et al., 2020). Above this, women might respond more sensitively to the overall experience of a psychological intervention, while the men tended to benefit on a day‐to‐day basis. Apart from that and in the context of depression, current depressive symptoms in the women predicted lower cortisol levels and a flatter recovery curve in response to couple conflict, whereas men's depressive symptoms predicted higher cortisol levels (Powers et al., 2016). The type of stressor or salience, as well as the valence of an intervention might play a role in the different degrees of responsiveness: Overall, our finding is consistent with the general evidence of gender‐specific effects of HPA reactivity (Laurent et al., 2013) in contrast to sympathetic effects, which were largely missing in the present study.

The latter highlight the importance of considering psychobiological proxies of health as important indicators for the effectiveness of psychological interventions. This is in line with the RDoC to gain more sufficient insights into the interplay between psychological and biological processes (Insel et al., 2010; National Institute of Mental Health, 2023) and how they are linked in daily life. The current burgeoning of psychobiological indicators as evaluation markers, while still in its infancy, is indicative of a necessary broadening of the understanding of intervention effects on various health outcomes. Adding everyday life measures stimulates not only the connection between therapeutic gains of therapy sessions and patients' everyday lives, but also the sustainability of a broad range of *bio‐psycho‐social* intervention effects, which will play an increasingly important role in future studies.

The current findings must be considered in light of some limitations. First and foremost, the pattern of results is highly complex and indicates that the level of analyses (e.g., between‐dyad vs. within‐participant) and the biological sex of the partner are of relevance, not only on their own but also in various combinations. Considering the sex‐specific effects and in light of possible implications for personalized treatment options (e.g., different interventions for men and women), it seems necessary that future studies, with a higher statistical power, confirm these results. Likewise, it was not possible to include a fourth level and to explicitly consider possible dependencies of measurements within days (treating the data as nested in days and by including a respective random intercept).

Further, the results are based on a relatively homogenous Swiss sample including couples who were overall happy and healthy. In comparison, our previous data in couples with a female partner suffering from depression (Warth et al., 2020) suggest that the PAT might not have the same effects in clinical populations. For instance, the attribution of positive interaction behaviour depends on the relationship quality: Happy couples are more likely to attribute the partners' positive expression or behaviour as a partner trait, whereas negative interactions are more likely interpreted as a situational aspect. In the case of unhappy couples, it is exactly the opposite (Karney & Bradbury, 1995), which is important to consider for the application of such minimal interventions in the clinical context and in malfunctioning couples. Nevertheless, for example, in stepped‐care approaches where low‐threshold brief interventions are of particular importance from a public health perspective (e.g., Smith & Baucom, 2017), the PAT might serve as an easy‐to‐use tool with possible health‐relevant impact. Notably, most recent brain imaging data on compliment sharing in couples suggest that *both* the sender and the receiver of compliments experience increased reward‐related brain activation during the exchange of positive attributes (Eckstein et al., 2023). This might motivate couples to keep practising the PAT, particularly when they experienced some positive effects themselves and in their relationship.

Furthermore, low‐income couples tend to face an increased number of contextual stressors, which might also be considered when designing and applying couple interventions for daily life. In the present study, the study sample was very homogeneous regarding socio‐economic status. Thus, the question of who benefits most from which type and approach of interventions still remains unclear and needs to be addressed in future studies. Cultural differences are of further interest, which was for example addressed in a recent study focusing on the capitalization and relationship satisfaction in an Asian sample (De Netto et al., 2021). In addition, the current study only included mixed‐gender couples, whereas sexual and gender minority partners face specific stressors. The present results cannot be generalized easily and require elaborate investigations in future studies advancing our understanding of their differences and commonalities.

Lastly, in the present study, participants reported whether they adhered to the PAT or not daily, and not from moment to moment during their everyday life routines. Above this, with limiting the practising time to ‘up to two times’ during the assessment period, we might have limited variance in the data, and actually, the effect of the intervention. Future studies could aim at investigating the effects of a *momentary* PAT‐intervention (i.e., as an ecological momentary intervention) on individual and momentary stress levels.

### Summary and conclusions

These findings provide initial support that, indeed, individual health‐related outcomes, namely, perceived stress and biological stress‐sensitive markers might be directly addressed and improved with a minimal standard couple intervention. To verbally exchange positive aspects of each other and the romantic relationship up to three times per week (altogether up to 30 mins in total) could be associated with reduced individual stress levels, sCort, and sAA. Overall, the findings offer translation of epidemiological findings about relationship quality and individual health to psychological interventions with potential long‐term health impact. Notably, the intervention designed and used here does not provide standard phrases or automated positive feedback but the dyadically developed focus on specific positive aspects within the own romantic relationship. Here, in genuinely happy couples, the PAT might serve as a specific strategy for improving couple interactions in daily life, employing positive reward and emphasizing shared appreciation for each other. Future research will need to translate these findings to larger and more diverse samples or clinical groups with different psychopathology affecting the couple relationship. Based on these results, other formats of implementation (e.g., automated feedback or using the partner's messages as ecological momentary intervention) and their effects on psychobiological outcomes and long‐term mental health indicators might be developed. Eventually, such dyadic interventions might augment psychological treatments by systematically involving the partner and building on the couple relationship as a resource for individual mental and physical health.

## AUTHOR CONTRIBUTIONS

**Corina Aguilar‐Raab:** Writing – original draft; writing – review and editing; visualization; formal analysis; methodology. **Martin Stoffel:** Writing – original draft; methodology; visualization; writing – review and editing; formal analysis. **Melanie Fischer:** Writing – review and editing. **Monika Eckstein:** Writing – review and editing. **Guy Bodenmann:** Conceptualization; methodology; writing – review and editing. **Markus Heinrichs:** Conceptualization; methodology; writing – review and editing. **Ulrike Ehlert:** Conceptualization; methodology; writing – review and editing. **Beate Ditzen:** Conceptualization; investigation; funding acquisition; methodology; writing – review and editing; supervision; resources; validation; formal analysis.

## CONFLICTS OF INTEREST

The authors declare that they have no conflict of interest.

## ETHICS STATEMENT

The study protocol was approved by the local ethics committee of the Canton of Zurich, swissmedics, and the study was monitored by the Clinical Trials Center Zurich. The research was conducted in accordance with the Declaration of Helsinki.

## CONSENT TO PARTICIPATE

All individual participants provided their written informed consent to participate in this study.

## Supporting information


Data S1:

Data S2:

Data S3:

## Data Availability

The data that support the findings of this study are openly available in Heidata at https://heidata.uni‐heidelberg.de/.

## Footnotes

^1^
Missing data for sCort and sAA was imputed on an individual level with the algorithm to impute single missing values from measures 1, 2 and 6 with the mean of this measure from the other days. Single missing data from measures 3, 4 and 5 were imputed by interpolating that very day's previous and following measure. This resulted in missing data, if two or more samples were missing.

^2^
In case the data were transformed, outlier analyses were conducted after transformation.

^3^
Note that the predictor is on L3, since it is constant within each dyad, and therefore predicts between‐dyad differences, and not differences between persons.

^4^
This result is supported by a significant cross‐level interaction of sex‐by‐PAT; see the results of the sex‐specific analyses above for details.
