# Supplementary material for: Minimal couple intervention to improve psychobiological stress resilience
Source: Br J Health Psychol. 2025 May 13;30(2):e12799. doi: 10.1111/bjhp.12799 (PMC12070146; doi:10.1111/bjhp.12799)
Supplement: Supplementary file 1 — Data S1: [file BJHP-30-0-s003.docx]

**Supplement 1**

**PARTNERSHIP APPRECIATION TASK (PAT): LIST OF POSITIVE ATTRIBUTES/THEMES**

In the following list, you can find attributes/themes that can arouse positive feelings in a partnership. Please look through the **items that are relevant to you** and look for additional attributes which you perceive as positive in your partnership, or where you have made positive experiences recently.

Please be **appreciative of your partner regarding the chosen attributes/themes** and focus on specific behaviour he/she has been showing in the past two months. If nothing more occurs to you regarding the chosen attributes/themes, try to remember beautiful moments in your partnership and tell your partner what affected you most about these.

| 1. Partner’s attention and emotional openness |
| --- |
| 2. Satisfaction with career |
| 3. Sharing of household work |
| 4. Shared beliefs about parenting |
| 5. Shared leisure time |
| 6. Friends and acquaintances |
| 7. Partner’s patience |
| 8. Peace of mind with regard to financial situation |
| 9. Attractiveness |
| 10. Trust |
| 11. Attachment |
| 12. Giving personal freedom |
| 13. Sexuality |
| 14. Loyalty |
| 15. Family / relatives |
| 16. Tolerance of each other’s personal habits |
| 17. Communication / joint discussions |
| 18. Desire to have children / family planning |
| 19. Supporting each other in everyday life matters |
| 20. Generosity |
| 21. Health / plans for the future |
| 22. Mutual support in times of crisis |
| 23. Tenderness |
| Others: |
