# Supplementary material for: Minimal couple intervention to improve psychobiological stress resilience
Source: Br J Health Psychol. 2025 May 13;30(2):e12799. doi: 10.1111/bjhp.12799 (PMC12070146; doi:10.1111/bjhp.12799)
Supplement: Supplementary file 2 — Data S2: [file BJHP-30-0-s001.docx]

**Supplement 2**

| **Table S1.** Perceived stress as a function of group assignment (PAT/nPAT). | | | | |
| --- | --- | --- | --- | --- |
|  |  | Fixed Effects | | |
|  |  | Estimates (se) |  | *p* |
| Intercept |  | 1.201 (.143) |  | < .001** |
| Time (linear) |  | .003 (.001) |  | < .001** |
| Time (quadratic) |  | -.000 (.000) |  | < .001** |
| Time (cubic) |  | .000 (.000) |  | < .001** |
| Age (years) |  | -.003 (.010) |  | .7924 |
| Sex (female / male) |  | .158 (.101) |  | .1272 |
| PAT/nPAT *** |  | .198 (.110) |  | .0801 |
|  |  | Random Effects (*SD*) | | |
| Level 3 (across dyads) |  |  |  |  |
| Intercept |  | .131 |  |  |
| Level 2 (across individuals) |  |  |  |  |
| Intercept |  | .392 |  |  |
| Residual |  | .812 |  |  |
| *Note.* Table depicts point estimates (standard errors for fixed effects in brackets). Covariances between random effects within a level were estimated (unstructured random effect matrices). Number of dyads = 40; Number of participants = 76; total number of observations = 1482. **p* < .05; ***p* < .001; *** Group assignment, treated as factor with nPAT (control group) and PAT (intervention group). Thirteen outliers were removed due to being beyond three SDs of the mean. Variable descriptions see *Note* of Table 1. | | | | |

| **Table S2.** Logarithmical salivary cortisol concentrations as a function of group assignment (PAT/nPAT). | | | | |
| --- | --- | --- | --- | --- |
|  |  | Fixed Effects | | |
|  |  | Estimates (se) |  | *p* |
| Intercept |  | 2.060 (.148) |  | < .001** |
| Time (linear) |  | .001 (.001) |  | .4282 |
| Time (quadratic) |  | -.000 (.000) |  | < .001** |
| Time (cubic) |  | .000 (.000) |  | < .001** |
| Intake of meal |  | -.000 (.009) |  | .9668 |
| Intake of drink |  | .012 (.013) |  | .3518 |
| Day activity |  | .022 (.011) |  | .0474* |
| Intake of caffeine |  | .023 (.047) |  | .6286 |
| Sleep quality |  | -.051(.013) |  | < .001** |
| Sleep problems |  | -.151 (.075) |  | .0436* |
| Age (years) |  | .004 (.010) |  | .7257 |
| Sex (female/ male) |  | .051 (.118) |  | .6690 |
| Body-mass-index (kg/m^2^) |  | .011 (.024) |  | .6442 |
| Hormonal contraceptives |  | .277 (.121) |  | .0296* |
| PAT/nPAT *** |  | -.197 (.126) |  | .1253 |
|  |  | Random Effects (*SD*) | | |
| Level 3 (across dyads) |  |  |  |  |
| Intercept |  | .287 |  |  |
| Time (linear) |  | .001 |  |  |
| Level 2 (across individuals) |  |  |  |  |
| Intercept |  | .241 |  |  |
| Residual |  | .683 |  |  |
| *Note.* Table depicts point estimates (standard errors for fixed effects in brackets). Covariances between random effects within a level were estimated (unstructured random effect matrices). Number of dyads = 40; Number of participants = 76; total number of observations = 1461. **p* < .05; ***p* < .001; *** Group assignment, treated as factor with nPAT (control group) and PAT (intervention group). Ten outliers were removed due to being beyond three SDs of the mean. Variable descriptions see *Note* of Table 1. | | | | |

| **Table S3.** Logarithmical salivary alpha-amylase concentrations as a function of group assignment (PAT/nPAT). | | | | |
| --- | --- | --- | --- | --- |
|  |  | Fixed Effects | | |
|  |  | Estimates (se) |  | *p* |
| Intercept |  | 3.892 (.181) |  | < .001** |
| Time (linear) |  | .002 (.000) |  | < .001** |
| Time (quadratic) |  | -.000 (.000) |  | < .001** |
| Intake of meal |  | .031 (.009) |  | < .001** |
| Intake of drink |  | -.002 (.013) |  | .8780 |
| Day activity |  | .014 (.011) |  | .2073 |
| Intake of caffeine |  | .068 (.047) |  | .1527 |
| Sleep quality |  | -.016 (.013) |  | .2188 |
| Sleep problems |  | -.040 (.078) |  | .6075 |
| Age (years) |  | .003 (.022) |  | .8929 |
| Sex (female/ male) |  | -.262 (.294) |  | .3801 |
| Body-mass-index (kg/m^2^) |  | .050 (.057) |  | .3881 |
| Hormonal contraceptives |  | .358 (.298) |  | .2377 |
| PAT/nPAT *** |  | -.320 (.241) |  | .1912 |
|  |  | Random Effects (*SD*) | | |
| Level 3 (across dyads) |  |  |  |  |
| Intercept |  | .496 |  |  |
| Level 2 (across individuals) |  |  |  |  |
| Intercept |  | .747 |  |  |
| Residual |  | .695 |  |  |
| *Note.* Table depicts point estimates (standard errors for fixed effects in brackets). Covariances between random effects within a level were estimated (unstructured random effect matrices). Number of dyads = 40; Number of participants = 76; total number of observations = 1456. **p* < .05; ***p* < .001; *** Group assignment, treated as factor with nPAT (control group) and PAT (intervention group). Seven outliers were removed due to being beyond three SDs of the mean. Variable descriptions see *Note* of Table 1. | | | | |

| **Table S4.** Perceived stress as a function of group assignment (PAT/nPAT). | | | | | | | |
| --- | --- | --- | --- | --- | --- | --- | --- |
|  |  | Fixed Effects | | | | | |
|  |  | Men | | | Women | | |
|  |  | Estimates (se) |  | *p* | Estimates (se) |  | *p* |
| Intercept |  | 1.484 (.116) |  | < .001** | 2.020 (.123) |  | < .001** |
| Time (linear) |  | -.006 (.000) |  | < .001** | -.001 (.000) |  | < .001** |
| Age (years) |  | .002 (.014) |  | 0.8855 | -.007 (.014) |  | 0.6244 |
| PAT/nPAT *** |  | -.037 (.150) |  | 0.8062 | -.380 (.147) |  | 0.0098* |
|  |  | Random Effects (*SD*) | | | | | |
| Level 2 (across dyads) |  |  |  |  |  |  |  |
| Intercept |  | .434 |  |  | .382 |  |  |
| Residual |  | .752 |  |  | .884 |  |  |
| *Note.* Table depicts point estimates (standard errors for fixed effects in brackets). Covariances between random effects within a level were estimated (unstructured random effect matrices). Number of dyads = 40; total number of observations = 1482. **p* < .05; ***p* < .001; *** Group assignment, with 0 = nPAT (control group) and 1 = PAT (intervention group). Thirteen outliers were removed due to being beyond three SDs of the mean. Variable descriptions see *Note* of Table 1. | | | | | | | |

| **Table S5.** Logarithmical salivary cortisol concentrations as a function of group assignment (PAT/nPAT). | | | | | | | |
| --- | --- | --- | --- | --- | --- | --- | --- |
|  |  | Fixed Effects | | | | | |
|  |  | Men | | | Women | | |
|  |  | Estimates (se) |  | *p* | Estimates (se) |  | *p* |
| Intercept |  | 1.713 (.206) |  | < .001** | 2.355 (.201) |  | < .001** |
| Time (linear) |  | .002 (.001) |  | .0794 | -.000 (.001) |  | .6791 |
| Time (quadratic) |  | -.000 (.000) |  | < .001** | -.000 (.000) |  | .0335* |
| Time (cubic) |  | .000 (.000) |  | < .001** | .000 (.000) |  | .0041* |
| Intake of meal |  | -.007 (.012) |  | .5624 | .010 (.012) |  | .3805 |
| Intake of drink |  | .019 (.019) |  | .3211 | -.004 (.018) |  | .8318 |
| Day activity |  | .022 (.016) |  | .1668 | .012 (.015) |  | .4208 |
| Intake of caffeine |  | .035 (.067) |  | .6040 | .012 (.064) |  | .8472 |
| Sleep quality |  | -.047 (.019) |  | .0123* | -.050 (.016) |  | .0019* |
| Sleep problems |  | -.489 (.134) |  | < .001** | .016 (.086) |  | .8495 |
| Age (years) |  | .012 (.012) |  | .3256 | -.009 (.014) |  | .5397 |
| Body-mass-index (kg/m^2^) |  | -.010 (.036) |  | .7788 | .012 (.035) |  | .7396 |
| PAT/nPAT *** |  | -.207 (.133) |  | .1200 | -.188 (.154) |  | .2232 |
|  |  | Random Effects (*SD*) | | | | | |
| Level 2 (across dyads) |  |  |  |  |  |  |  |
| Intercept |  | .394 |  |  | .387 |  |  |
| Time (linear) |  | .001 |  |  | .001 |  |  |
| Residual |  | .717 |  |  | .617 |  |  |
| *Note.* Table depicts point estimates (standard errors for fixed effects in brackets). Covariances between random effects within a level were estimated (unstructured random effect matrices). Number of dyads = 40; total number of observations = 1461. **p* < .05; ***p* < .001; *** Group assignment, with 0 = nPAT (control group) and 1 = PAT (intervention group). Ten outliers were removed due to being beyond three SDs of the mean. Variable descriptions see *Note* of Table 1. | | | | | | | |

| **Table S6.** Logarithmical salivary alpha-amylase concentrations as a function of group assignment (PAT/nPAT). | | | | | | | |
| --- | --- | --- | --- | --- | --- | --- | --- |
|  |  | Fixed Effects | | | | | |
|  |  | Men | | | Women | | |
|  |  | Estimates (se) |  | *p* | Estimates (se) |  | *p* |
| Intercept |  | 4.042 (.263) |  | < .001** | 4.052 (.256) |  | < .001** |
| Time (linear) |  | .002 (.000) |  | < .001** | .002 (.000) |  | < .001** |
| Time (quadratic) |  | -.000 (.000) |  | < .001** | -.000 (.000) |  | < .001** |
| Intake of meal |  | .045 (.012) |  | < .001** | .016 (.013) |  | .2442 |
| Intake of drink |  | .013 (.018) |  | .4596 | -.015 (.020) |  | .4453 |
| Day activity |  | .013 (.015) |  | .3972 | .019 (.017) |  | .2504 |
| Intake of caffeine |  | .067 (.064) |  | .2957 | .071 (.072) |  | .3228 |
| Sleep quality |  | .006 (.019) |  | .7476 | -.037 (.019) |  | .0532 |
| Sleep problems |  | -.159 (.127) |  | .2109 | .014 (.099) |  | .8874 |
| Age (years) |  | -.001 (.029) |  | .9760 | .007 (.029) |  | .8195 |
| Body-mass-index (kg/m^2^) |  | -.043 (.090) |  | .6371 | .126 (.072) |  | .0809 |
| PAT/nPAT *** |  | -.177 (.303) |  | .5586 | -.527 (.294) |  | .0729 |
|  |  | Random Effects (*SD*) | | | | | |
| Level 2 (across dyads) |  |  |  |  |  |  |  |
| Intercept |  | .926 |  |  | .843 |  |  |
| Residual |  | .683 |  |  | .704 |  |  |
| *Note.* Table depicts point estimates (standard errors for fixed effects in brackets). Covariances between random effects within a level were estimated (unstructured random effect matrices). Number of dyads = 40; total number of observations = 1456. **p* < .05; ***p* < .001; *** Group assignment, with 0 = nPAT (control group) and 1 = PAT (intervention group). Seven outliers were removed due to being beyond three SDs of the mean. Variable descriptions see *Note* of Table 1. | | | | | | | |

| **Table S7.** Perceived stress as a function of practising the PAT | | | | |
| --- | --- | --- | --- | --- |
|  |  | Fixed Effects | | |
|  |  | Estimates (se) |  | *p* |
| Intercept |  | 1.033 (.355) |  | .0037* |
| Time (linear) |  | .000 (.000) |  | .3290 |
| Time (quadratic) |  | -.000 (.000) |  | .0023* |
| Age (years) |  | .006 (.031) |  | .8462 |
| Sex (female / male) |  | -.005 (.151) |  | .9732 |
| Person mean of positive interactions (practicing the PAT)^a^ |  | 1.000 (.932) |  | .3003 |
| Positive interaction (practicing the PAT) *** |  | -.184 (.106) |  | .0834 |
|  |  | Random Effects (*SD*) | | |
| Level 3 (across dyads) |  |  |  |  |
| Intercept |  | .303 |  |  |
| Positive interaction (practicing the PAT) |  | .370 |  |  |
| Level 2 (across individuals) |  |  |  |  |
| Intercept |  | .406 |  |  |
| Residual |  | .780 |  |  |
| *Note.* Table depicts point estimates (standard errors for fixed effects in brackets). Covariances between random effects within a level were estimated (unstructured random effect matrices). Number of dyads = 19; Number of participants = 37; total number of observations = 709. **p* < .05; ***p* < .001; ***Positive interaction in everyday life (treated as factor; no / yes). Four outliers were removed due to being beyond three SDs of the mean. Variable descriptions see *Note* of Table 1.^a^Contextual effect of practicing the PAT. | | | | |

| **Table S8.** Logarithmical salivary cortisol concentrations as a function of practising the PAT | | | | |
| --- | --- | --- | --- | --- |
|  |  | Fixed Effects | | |
|  |  | Estimates (SE) |  | *p* |
| Intercept |  | 1.738 (.322) |  | < .001** |
| Time (linear) |  | .003 (.001) |  | .0140* |
| Time (quadratic) |  | -.000 (.000) |  | < .001** |
| Time (cubic) |  | .000 (.000) |  | < .001** |
| Intake of meal |  | -.016 (.013) |  | .2134 |
| Intake of drink |  | .019 (.020) |  | .3476 |
| Day activity |  | .022 (.016) |  | .1624 |
| Intake of caffeine |  | .021 (.068) |  | .7596 |
| Sleep quality |  | -.041 (.019) |  | .0281* |
| Sleep problems |  | -.083 (.096) |  | .3881 |
| Age (years) |  | -.009 (.024) |  | .7048 |
| Sex (female/ male) |  | .110 (.152) |  | .4842 |
| Body-mass-index (kg/m^2^) |  | .008 (.032) |  | .7995 |
| Hormonal contraceptives |  | .149 (.179) |  | .4200 |
| Person mean of positive interactions (practicing the PAT)^a^ |  | -.305 (.734) |  | .6843 |
| Positive interaction (practicing the PAT) *** |  | -.127 (.054) |  | .0197* |
|  |  | Random Effects (*SD*) | | |
| Level 3 (across dyads) |  |  |  |  |
| Intercept |  | .318 |  |  |
| Time (linear) |  | .001 |  |  |
| Level 2 (across individuals) |  |  |  |  |
| Intercept |  | .259 |  |  |
| Residual |  | .669 |  |  |
| *Note.* Table depicts point estimates (standard errors for fixed effects in brackets). Covariances between random effects within a level were estimated (unstructured random effect matrices). Number of dyads = 19; Number of participants = 37; total number of observations = 700. **p* < .05; ***p* < .001; ***Positive interaction in everyday life (treated as factor; no / yes). Four outliers were removed due to being beyond three SDs of the mean*.* Variable descriptions see *Note* of Table 1. ^a^Contextual effect of practicing the PAT. | | | | |

| **Table S9.** Logarithmical salivary alpha-amylase concentrations as a function of practising the PAT | | | | |
| --- | --- | --- | --- | --- |
|  |  | Fixed Effects | | |
|  |  | Estimates (se) |  | *p* |
| Intercept |  | 2.726 (.589) |  | < .001** |
| Time (linear) |  | .002 (.000) |  | < .001** |
| Time (quadratic) |  | -.000 (.000) |  | < .001** |
| Intake of meal |  | .030 (.014) |  | .0321* |
| Intake of drink |  | -.016 (.021) |  | .4284 |
| Day activity |  | .027 (.017) |  | .1184 |
| Intake of caffeine |  | .007 (.071) |  | .9179 |
| Sleep quality |  | -.025 (.021) |  | .2207 |
| Sleep problems |  | -.056 (.105) |  | .5937 |
| Age (years) |  | -.019 (.053) |  | .7302 |
| Sex (female/ male) |  | -.387 (.368) |  | .3123 |
| Body-mass-index (kg/m^2^) |  | .040 (.072) |  | .5856 |
| Hormonal contraceptives |  | -.026 (.410) |  | .9497 |
| Person mean of positive interactions (practicing the PAT)^a^ |  | 2.396 (1.590) |  | .1556 |
| Positive interaction (practicing the PAT) *** |  | -.122 (.058) |  | .0369* |
|  |  | Random Effects (*SD*) | | |
| Level 3 (across dyads) |  |  |  |  |
| Intercept |  | .432 |  |  |
| Level 2 (across individuals) |  |  |  |  |
| Intercept |  | .732 |  |  |
| Residual |  | .719 |  |  |
| *Note.* Table depicts point estimates (standard errors for fixed effects in brackets). Covariances between random effects within a level were estimated (unstructured random effect matrices). Number of dyads = 19; Number of participants = 37; total number of observations = 699. **p* < .05; ***p* < .001; ***Positive interaction in everyday life (treated as factor; no / yes). One outlier was removed due to being beyond three SDs of the mean. Variable descriptions see *Note* of Table 1. ^a^Contextual effect of practicing the PAT. | | | | |

| **Table S10.** Perceived stress as a function of practising the PAT | | | | | | | |
| --- | --- | --- | --- | --- | --- | --- | --- |
|  |  | Fixed Effects | | | | | |
|  |  | Men | | | Women | | |
|  |  | Estimates (SE) |  | *p* | Estimates (SE) |  | *p* |
| Intercept |  | 1.453 (.453) |  | . 0014* | 1.009 (.442) |  | .0228* |
| Time (linear) |  | -.001 (.000) |  | < .001** | -.001 (.000) |  | < .001** |
| Age (years) |  | .039 (.040) |  | .3298 | -.045 (.042) |  | .2885 |
| Person mean of positive interactions (practicing the PAT)^a^ |  | .234 (1.258) |  | 0.8523 | 1.762 (1.141) |  | 0.1231 |
| Positive interaction (practicing the PAT) |  | -.271 (.082) |  | .0011* | -.074 (.098) |  | .4514 |
|  |  | Random Effects (*SD*) | | | | | |
| Level 2 (across dyads) |  |  |  |  |  |  |  |
| Intercept |  | .503 |  |  | .445 |  |  |
| Residual |  | .705 |  |  | .880 |  |  |
| *Note.* Table depicts point estimates (standard errors for fixed effects in brackets). Covariances between random effects within a level were estimated (unstructured random effect matrices). Number of dyads = 19; total number of observations = 709. **p* < .05; ***p* < .001; ***Positive interaction in everyday life. Four outliers were removed due to being beyond three SDs of the mean. Variable descriptions see *Note* of Table 1. ^a^Contextual effect of practicing the PAT. | | | | | | | |

| **Table S11.** Logarithmical salivary cortisol concentrations as a function of practising the PAT | | | | | | | |
| --- | --- | --- | --- | --- | --- | --- | --- |
|  |  | Fixed Effects | | | | | |
|  |  | Men | | | Women | | |
|  |  | Estimates (SE) |  | *p* | Estimates (SE) |  | *p* |
| Intercept |  | 1.570 (.463) |  | < .001** | 1.844 (.371) |  | < .001** |
| Time (linear) |  | .004 (.002) |  | .0449* | .002 (.002) |  | .1165 |
| Time (quadratic) |  | -.000 (.000) |  | < .001** | -.000 (.000) |  | < .001** |
| Time (cubic) |  | .000 (.000) |  | < .001** | .000 (.000) |  | < .001** |
| Intake of meal |  | -.002 (.019) |  | .9088 | -.020 (.018) |  | .2687 |
| Intake of drink |  | -.004 (.029) |  | .8977 | .022 (.026) |  | .4049 |
| Day activity |  | .045 (.025) |  | .0731 | .005 (.020) |  | .7968 |
| Intake of caffeine |  | -.004 (.102) |  | .9703 | .033 (.091) |  | .7145 |
| Sleep quality |  | -.024 (.030) |  | .4119 | -.056 (.022) |  | .0111* |
| Sleep problems |  | -.183 (.176) |  | .2989 | -.054 (.110) |  | .6236 |
| Age (years) |  | .015 (.034) |  | .6613 | -.032 (.026) |  | .2277 |
| Body-mass-index (kg/m^2^) |  | -.045 (.054) |  | .4077 | .030 (.032) |  | .3563 |
| Person mean of positive interactions (practicing the PAT)^a^ |  | -.214 (1.082) |  | 0.8430 | -.100 (.715) |  | 0.8937 |
| Positive interaction (practicing the PAT) |  | -.226 (.082) |  | .0057 | -.013 (.070) |  | .8524 |
|  |  | Random Effects (*SD*) | | | | | |
| Level 2 (across dyads) |  |  |  |  |  |  |  |
| Intercept |  | .566 |  |  | .350 |  |  |
| Time (linear) |  | .001 |  |  | .001 |  |  |
| Residual |  | .680 |  |  | .619 |  |  |
| *Note.* Table depicts point estimates (standard errors for fixed effects in brackets). Covariances between random effects within a level were estimated (unstructured random effect matrices). Number of dyads = 19; total number of observations = 700. **p* < .05; ***p* < .001; ***Positive interaction in everyday life. Four outliers were removed due to being beyond three SDs of the mean*.* Variable descriptions see *Note* of Table 1. ^a^Contextual effect of practicing the PAT. | | | | | | | |

| **Table S12.** Logarithmical salivary alpha-amylase concentrations as a function of practising the PAT | | | | | | | |
| --- | --- | --- | --- | --- | --- | --- | --- |
|  |  | Fixed Effects | | | | | |
|  |  | Men | | | Women | | |
|  |  | Estimates (SE) |  | *p* | Estimates (SE) |  | *p* |
| Intercept |  | 4.094 (.727) |  | < .001** | 1.588 (.776) |  | .0412* |
| Time (linear) |  | .002 (.001) |  | .0058* | .002 (.001) |  | < .001** |
| Time (quadratic) |  | -.000 (.000) |  | . < .001** | -.000 (.000) |  | < .001** |
| Intake of meal |  | .037 (.020) |  | .9088 | .022 (.019) |  | .2555 |
| Intake of drink |  | .015 (.030) |  | .6133 | -.047 (.028) |  | .1005 |
| Day activity |  | .037 (.026) |  | .1576 | .015 (.022) |  | .4814 |
| Intake of caffeine |  | -.031 (.107) |  | .7688 | .032 (.097) |  | .7436 |
| Sleep quality |  | -.031 (.034) |  | .3603 | -.024 (.026) |  | .3527 |
| Sleep problems |  | -.176 (.191) |  | .3588 | -.019 (.124) |  | .8801 |
| Age (years) |  | -.054 (.063) |  | .3885 | .001 (.072) |  | .9901 |
| Body-mass-index (kg/m^2^) |  | .022 (.101) |  | .8282 | .068 (.088) |  | .4450 |
| Person mean of positive interactions (practicing the PAT)^a^ |  | -.6703 (2.010) |  | .7643 | 4.843 (1.929) |  | 0.0123* |
| Positive interaction (practicing the PAT) |  | -.072 (.090) |  | .4278 | -.145 (.078) |  | .0645 |
|  |  | Random Effects (*SD*) | | | | | |
| Level 2 (across dyads) |  |  |  |  |  |  |  |
| Intercept |  | .834 |  |  | .812 |  |  |
| Residual |  | .749 |  |  | .688 |  |  |
| *Note.* Table depicts point estimates (standard errors for fixed effects in brackets). Covariances between random effects within a level were estimated (unstructured random effect matrices). Number of dyads = 19; total number of observations = 699. **p* < .05; ***p* < .001; ***Positive interaction in everyday life. One outlier was removed due to being beyond three SDs of the mean. Variable descriptions see *Note* of Table 1. ^a^Contextual effect of practicing the PAT. | | | | | | | |
