# Supplementary material for: Minimal couple intervention to improve psychobiological stress resilience
Source: Br J Health Psychol. 2025 May 13;30(2):e12799. doi: 10.1111/bjhp.12799 (PMC12070146; doi:10.1111/bjhp.12799)
Supplement: Supplementary file 3 — Data S3: [file BJHP-30-0-s002.docx]

**Supplement 3**

**Model 1: Effect of group assignment on perceived stress**

| Level 1 (measurement occasion level): |  |
| --- | --- |
| ${stress}_{idj}=\beta_{0dj}+\beta_{1}{(time}_{idj})+\beta_{2}{(time^{2}}_{idj})+ \beta_{3}{(time^{3}}_{idj})+\varepsilon_{idj}$ | (1) |
|  |  |

| Level 2 (person level): |  |
| --- | --- |
| $\beta_{0dj}=\gamma_{00j}+\gamma_{01}{(age}_{dj})+\gamma_{02}{(sex}_{dj})+ \zeta_{0dj}$ | (2) |
|  |  |

| Level 3 (dyad level): |  |
| --- | --- |
| $\gamma_{00j}=\pi_{00}+ \pi_{01}{(PAT/nPAT}_{j})+\upsilon_{0j}$ | (3) |
|  |  |

**Model 2: Effect of group assignment on sCort**

| Level 1 (measurement occasion level): |  |
| --- | --- |
| ${sCort}_{idj}=\beta_{0dj}+\beta_{1j}{(time}_{idj})+\beta_{2}{(time^{2}}_{idj})+ {\beta_{3}{(time^{3}}_{idj})+ \beta}_{4}{(food}_{idj})+\beta_{5}{(drink}_{idj})+\beta_{6}{(activity}_{idj})+\beta_{7}{(caffeine}_{idj})+\beta_{8}{(slp.quality}_{idj})+ \beta_{9}{(slp.problems}_{idj})+ \varepsilon_{idj}$ | (1) |
|  |  |

| Level 2 (person level): |  |
| --- | --- |
| $\beta_{0dj}=\gamma_{00j}+\gamma_{01}{(age}_{dj})$  $+\gamma_{02}{(sex}_{dj})+\gamma_{03}{(bmi}_{dj})+\gamma_{04}{(contraceptives}_{dj})+ \zeta_{0dj}$ | (2) |
|  |  |

| Level 3 (dyad level): |  |
| --- | --- |
| $\gamma_{00j}=\pi_{00}+ \pi_{01}{(PAT/nPAT}_{j})+\upsilon_{0j}$ | (3) |
| $\beta_{1j}=\pi_{10}+\upsilon_{1j}$ | (4) |
|  |  |

**Model 3: Effect of group assignment on sAA**

| Level 1 (measurement occasion level): |  |
| --- | --- |
| ${sAA}_{idj}=\beta_{0dj}+\beta_{1}{(time}_{idj})+\beta_{2}{(time^{2}}_{idj})+ \beta_{3}{(food}_{idj})+\beta_{4}{(drink}_{idj})+\beta_{5}{(activity}_{idj})+\beta_{6}{(caffeine}_{idj})+\beta_{7}{(slp.quality}_{idj})+ \beta_{8}{(slp.problems}_{idj})+ \varepsilon_{idj}$ | (1) |
|  |  |

| Level 2 (person level): |  |
| --- | --- |
| $\beta_{0dj}=\gamma_{00j}+\gamma_{01}{(age}_{dj})$  $+\gamma_{02}{(sex}_{dj})+\gamma_{03}{(bmi}_{dj})+\gamma_{04}{(contraceptives}_{dj})+ \zeta_{0dj}$ | (2) |
|  |  |

| Level 3 (dyad level): |  |
| --- | --- |
| $\gamma_{00j}=\pi_{00}+ \pi_{01}{(PAT/nPAT}_{j})+\upsilon_{0j}$ | (3) |
|  |  |

**Model 4: Effect of group assignment (PAT/nPAT) on perceived stress** (subscripts “M” and “F” indicate estimates for “males” and “females”, respectively)

| Level 1 (measurement occasion level): |  |
| --- | --- |
| ${stress}_{ij}=\beta_{0jM}+\beta_{1M}{(time}_{ij})+ \varepsilon_{ijM}+$  $\beta_{0jF}+\beta_{1F}{(time}_{ij}) {+ \varepsilon}_{ijF}$ | (1) |
| Level 2 (dyad level): |  |
| $\beta_{0jM}=\gamma_{00M}+\gamma_{01M}({PAT/nPAT}_{j})+\gamma_{02M}({age}_{j})+ \zeta_{0jM}$ | (2) |
| $\beta_{0jF}=\gamma_{00F}+\gamma_{01F}({PAT/nPAT}_{j})+\gamma_{02F}({age}_{j})+ \zeta_{0jF}$ | (3) |

**Model 5: Effect of group assignment (PAT/nPAT) on sCort** (subscripts “M” and “F” indicate estimates for “males” and “females”, respectively)

| Level 1 (measurement occasion level): |  |
| --- | --- |
| ${sCort}_{ij}=\beta_{0jM}+\beta_{1jM}{(time}_{ij})+ {\beta_{2M}{(time^{2}}_{ij})+\beta_{3M}{(time^{3}}_{ij})+\beta_{4M}\left( {food}_{ij} \right)+\beta_{5M}\left( {drink}_{ij} \right)+\beta_{6M}\left( {activity}_{ij} \right)+\beta_{7M}\left( {caffeine}_{ij} \right)+\beta_{8M}\left( {slp.quality}_{ij} \right)+\beta_{9M}\left( {slp.problems}_{ij} \right)+ \varepsilon}_{ijM}+$  $\beta_{0jF}+\beta_{1jF}{(time}_{ij})+ {\beta_{2F}{(time^{2}}_{ij})+\beta_{3F}{(time^{3}}_{ij})+\beta_{4F}\left( {food}_{ij} \right)+\beta_{5F}\left( {drink}_{ij} \right)+\beta_{6F}\left( {activity}_{ij} \right)+\beta_{7F}\left( {caffeine}_{ij} \right)+\beta_{8F}\left( {slp.quality}_{ij} \right)+\beta_{9F}\left( {slp.problems}_{ij} \right)+ \varepsilon}_{ijF}$ | (1) |
| Level 2 (dyad level): |  |
| $\beta_{0jM}=\gamma_{00M}+\gamma_{01M}({PAT/nPAT}_{j})+\gamma_{02M}({age}_{j})+\gamma_{03M}({bmi}_{j})+\zeta_{0jM}$ | (2) |
| $\beta_{1jM}=\pi_{10M}+\upsilon_{1jM}$ | (3) |
| $\beta_{0jM}=\gamma_{00F}+\gamma_{01F}({PAT/nPAT}_{j})+\gamma_{02F}({age}_{j})+\gamma_{03F}({bmi}_{j})+\zeta_{0jF}$ | (4) |
| $\beta_{1jF}=\pi_{10F}+\upsilon_{1jF}$ | (5) |

**Model 6: Effect of group assignment (PAT/nPAT) on sAA** (subscripts “M” and “F” indicate estimates for “males” and “females”, respectively)

| Level 1 (measurement occasion level): |  |
| --- | --- |
| ${sAA}_{ij}=\beta_{0jM}+\beta_{1M}{(time}_{ij})+ {\beta_{2M}{(time^{2}}_{ij})+\beta_{3M}\left( {food}_{ij} \right)+\beta_{4M}\left( {drink}_{ij} \right)+\beta_{5M}\left( {activity}_{ij} \right)+\beta_{6M}\left( {caffeine}_{ij} \right)+\beta_{7M}\left( {slp.quality}_{ij} \right)+\beta_{8M}\left( {slp.problems}_{ij} \right)+ \varepsilon}_{ijM}+$  $\beta_{0jF}+\beta_{1F}{(time}_{ij})+ {\beta_{2F}{(time^{2}}_{ij})+\beta_{3F}\left( {food}_{ij} \right)+\beta_{4F}\left( {drink}_{ij} \right)+\beta_{5F}\left( {activity}_{ij} \right)+\beta_{6F}\left( {caffeine}_{ij} \right)+\beta_{7F}\left( {slp.quality}_{ij} \right)+\beta_{8F}\left( {slp.problems}_{ij} \right)+ \varepsilon}_{ijF}$ | (1) |
| Level 2 (dyad level): |  |
| $\beta_{0jM}=\gamma_{00M}+\gamma_{01M}({PAT/nPAT}_{j})+\gamma_{02M}({age}_{j})+\gamma_{03M}({bmi}_{j})+\zeta_{0jM}$ | (2) |
| $\beta_{0jM}=\gamma_{00F}+\gamma_{01F}({PAT/nPAT}_{j})+\gamma_{02F}({age}_{j})+\gamma_{03F}({bmi}_{j})+\zeta_{0jF}$ | (3) |

**Model 7: Effect of practicing the PAT on perceived stress**

| Level 1 (measurement occasion level): |  |
| --- | --- |
| ${stress}_{idj}=\beta_{0dj}+\beta_{1}{(time}_{idj})+\beta_{2}{(time^{2}}_{idj})+ \beta_{3j}{(practicing the PAT}_{idj})+ \varepsilon_{idj}$ | (1) |
|  |  |

| Level 2 (person level): |  |
| --- | --- |
| $\beta_{0dj}=\gamma_{00j}+\gamma_{01}{(age}_{dj})$  $+\gamma_{02}{(sex}_{dj})+\gamma_{03}{(person mean practicing the PAT}_{dj}) + \zeta_{0dj}$ | (2) |
|  |  |

| Level 3 (dyad level): |  |
| --- | --- |
| $\gamma_{00j}=\pi_{00}+\upsilon_{0j}$ | (3) |
| $\beta_{3j}=\pi_{30}+\upsilon_{3j}$ | (4) |
|  |  |

**Model 8: Effect of practicing the PAT on sCort**

| Level 1 (measurement occasion level): |  |
| --- | --- |
| ${sCort}_{idj}=\beta_{0dj}+\beta_{1j}{(time}_{idj})+\beta_{2}{(time^{2}}_{idj})+ \beta_{3}{(time^{3}}_{idj})+ \beta_{4}{(food}_{idj})+\beta_{5}{(drink}_{idj})+\beta_{6}{(activity}_{idj})+\beta_{7}{(caffeine}_{idj})+\beta_{8}{(slp.quality}_{idj})+ \beta_{9}{(slp.problems}_{idj})+ \beta_{10}{(practicing the PAT}_{idj})+ \varepsilon_{idj}$ | (1) |
|  |  |

| Level 2 (person level): |  |
| --- | --- |
| $\beta_{0dj}=\gamma_{00j}+\gamma_{01}{(age}_{dj})$  $+\gamma_{02}{(sex}_{dj})+\gamma_{03}{(bmi}_{dj})+\gamma_{04}{(contraceptives}_{dj})+\gamma_{05}{(person mean practicing the PAT}_{dj}) + \zeta_{0dj}$ | (2) |
|  |  |

| Level 3 (dyad level): |  |
| --- | --- |
| $\gamma_{00j}=\pi_{00}+\upsilon_{0j}$ | (3) |
| $\beta_{1j}=\pi_{10}+\upsilon_{1j}$ | (4) |
|  |  |

**Model 9: Effect of practicing the PAT on sAA**

| Level 1 (measurement occasion level): |  |
| --- | --- |
| ${sAA}_{idj}=\beta_{0dj}+\beta_{1}{(time}_{idj})+\beta_{2}{(time^{2}}_{idj})+ \beta_{3}{(food}_{idj})+\beta_{4}{(drink}_{idj})+\beta_{5}{(activity}_{idj})+\beta_{6}{(caffeine}_{idj})+\beta_{7}{(slp.quality}_{idj})+\beta_{8}{(slp.problems}_{idj})+ \beta_{9}{(practicing the PAT}_{idj})+ \varepsilon_{idj}$ | (1) |
|  |  |

| Level 2 (person level): |  |
| --- | --- |
| $\beta_{0dj}=\gamma_{00j}+\gamma_{01}{(age}_{dj})$  $+\gamma_{02}{(sex}_{dj})+\gamma_{03}{(bmi}_{dj})+\gamma_{04}{(contraceptives}_{dj})+\gamma_{05}{(person mean practicing the PAT}_{dj}) + \zeta_{0dj}$ | (2) |
|  |  |

| Level 3 (dyad level): |  |
| --- | --- |
| $\gamma_{00j}=\pi_{00}+\upsilon_{0j}$ | (3) |
|  |  |

**Model 10: Effect of practicing the PAT on perceived stress** (subscripts “M” and “F” indicate estimates for “males” and “females”, respectively)

| Level 1 (measurement occasion level): |  |
| --- | --- |
| ${stress}_{ij}=\beta_{0jM}+\beta_{1M}{(time}_{ij}) {{+ \beta}_{2M}{(practicing the PAT}_{ij})+ \varepsilon}_{ijM}+$  $\beta_{0jF}+\beta_{1F}{(time}_{ij}) {{+ \beta}_{2F}{(practicing the PAT}_{ij})+\varepsilon}_{ijF}$ | (1) |
| Level 2 (dyad level): |  |
| $\beta_{0jM}=\gamma_{00M}+\gamma_{01M}({person mean practicing the PAT}_{j})+\gamma_{02M}({age}_{j})+ \zeta_{0jM}$ | (2) |
| $\beta_{0jF}=\gamma_{00F}+\gamma_{01F}({person mean practicing the PAT}_{j})+\gamma_{02F}({age}_{j})+ \zeta_{0jF}$ | (3) |

**Model 11: Effect of practicing the PAT on sCort** (subscripts “M” and “F” indicate estimates for “males” and “females”, respectively)

| Level 1 (measurement occasion level): |  |
| --- | --- |
| ${sCort}_{ij}=\beta_{0jM}+\beta_{1jM}{(time}_{ij})+ {\beta_{2M}{(time^{2}}_{ij})+\beta_{3M}{(time^{3}}_{ij})+\beta_{4M}\left( {food}_{ij} \right)+\beta_{5M}\left( {drink}_{ij} \right)+\beta_{6M}\left( {activity}_{ij} \right)+\beta_{7M}\left( {caffeine}_{ij} \right)+\beta_{8M}\left( {slp.quality}_{ij} \right)+\beta_{9M}\left( {slp.problems}_{ij} \right)+\beta_{10M}{(practicing the PAT}_{ij})+ \varepsilon}_{ijM}+$  $\beta_{0jF}+\beta_{1jF}{(time}_{ij})+ {\beta_{2F}{(time^{2}}_{ij})+\beta_{3F}{(time^{3}}_{ij})+\beta_{4F}\left( {food}_{ij} \right)+\beta_{5F}\left( {drink}_{ij} \right)+\beta_{6F}\left( {activity}_{ij} \right)+\beta_{7F}\left( {caffeine}_{ij} \right)+\beta_{8F}\left( {slp.quality}_{ij} \right)+\beta_{9F}\left( {slp.problems}_{ij} \right){+ \beta}_{10F}{(practicing the PAT}_{ij})+ \varepsilon}_{ijF}$ | (1) |
| Level 2 (dyad level): |  |
| $\beta_{0jM}=\gamma_{00M}+\gamma_{01M}({person mean practicing the PAT}_{j})+\gamma_{02M}({age}_{j})+\gamma_{03M}({bmi}_{j})+\zeta_{0jM}$ | (2) |
| $\beta_{1jM}=\pi_{10M}+\upsilon_{1jM}$ | (3) |
| $\beta_{0jM}=\gamma_{00F}+\gamma_{01F}({person mean practicing the PAT}_{j})+\gamma_{02F}({age}_{j})+\gamma_{03F}({bmi}_{j})+\zeta_{0jF}$ | (4) |
| $\beta_{1jF}=\pi_{10F}+\upsilon_{1jF}$ | (5) |

**Model 12: Effect of practicing the PAT on sAA** (subscripts “M” and “F” indicate estimates for “males” and “females”, respectively)

| Level 1 (measurement occasion level): |  |
| --- | --- |
| ${sAA}_{ij}=\beta_{0jM}+\beta_{1M}{(time}_{ij})+ {\beta_{2M}{(time^{2}}_{ij})+\beta_{3M}\left( {food}_{ij} \right)+\beta_{4M}\left( {drink}_{ij} \right)+\beta_{5M}\left( {activity}_{ij} \right)+\beta_{6M}\left( {caffeine}_{ij} \right)+\beta_{7M}\left( {slp.quality}_{ij} \right)+\beta_{8M}\left( {slp.problems}_{ij} \right)+\beta_{9M}{(practicing the PAT}_{ij})+ \varepsilon}_{ijM}+$  $\beta_{0jF}+\beta_{1F}{(time}_{ij})+ {\beta_{2F}{(time^{2}}_{ij})+\beta_{3F}\left( {food}_{ij} \right)+\beta_{4F}\left( {drink}_{ij} \right)+\beta_{5F}\left( {activity}_{ij} \right)+\beta_{6F}\left( {caffeine}_{ij} \right)+\beta_{7F}\left( {slp.quality}_{ij} \right)+\beta_{8F}\left( {slp.problems}_{ij} \right){+ \beta}_{9F}{(practicing the PAT}_{ij})+ \varepsilon}_{ijF}$ | (1) |
| Level 2 (dyad level): |  |
| $\beta_{0jM}=\gamma_{00M}+\gamma_{01M}({person mean practicing the PAT}_{j})+\gamma_{02M}({age}_{j})+\gamma_{03M}({bmi}_{j})+\zeta_{0jM}$ | (2) |
| $\beta_{0jM}=\gamma_{00F}+\gamma_{01F}({person mean practicing the PAT}_{j})+\gamma_{02F}({age}_{j})+\gamma_{03F}({bmi}_{j})+\zeta_{0jF}$ | (3) |
